# A pan‐cancer analysis reveals genetic alterations, molecular mechanisms, and clinical relevance of m^5^C regulators

**DOI:** 10.1002/ctm2.180

**Published:** 2020-09-15

**Authors:** E Du, Jingxian Li, Fei Sheng, Shuai Li, Jianqiang Zhu, Yong Xu, Zhihong Zhang

**Affiliations:** ^1^ Tianjin Institute of Urology The Second Hospital of Tianjin Medical University Tianjin China; ^2^ Department of Joint Tianjin Hospital No. 406 Jiefang South Rd, Hexi District Tianjin 300211 China

**Dear Editor,**

The RNA modification is determined by the coordinated actions of the three regulators: methyltransferases (writers), RNA‐binding proteins (readers), and demethylases (erasers).^1^ At present, m^5^C regulators consist of eight writers and two readers.^2^ The erasers of m^5^C have not yet been identified (Figure 1A). Current evidence suggests that m^5^C perturbations mediated by these regulators are involved in cell differentiation and apoptosis.^3^ Mutations in the genes encoding these regulators are linked with various human diseases, and the changes in expression levels have been observed in numerous cancers.^4^ However, the roles of m^5^C regulators in cancers still remain ambiguous. So it is valuable to investigate the genetic alterations and functional disorders of m^5^C regulators for therapeutic targets in multiple cancers.

**FIGURE 1:**
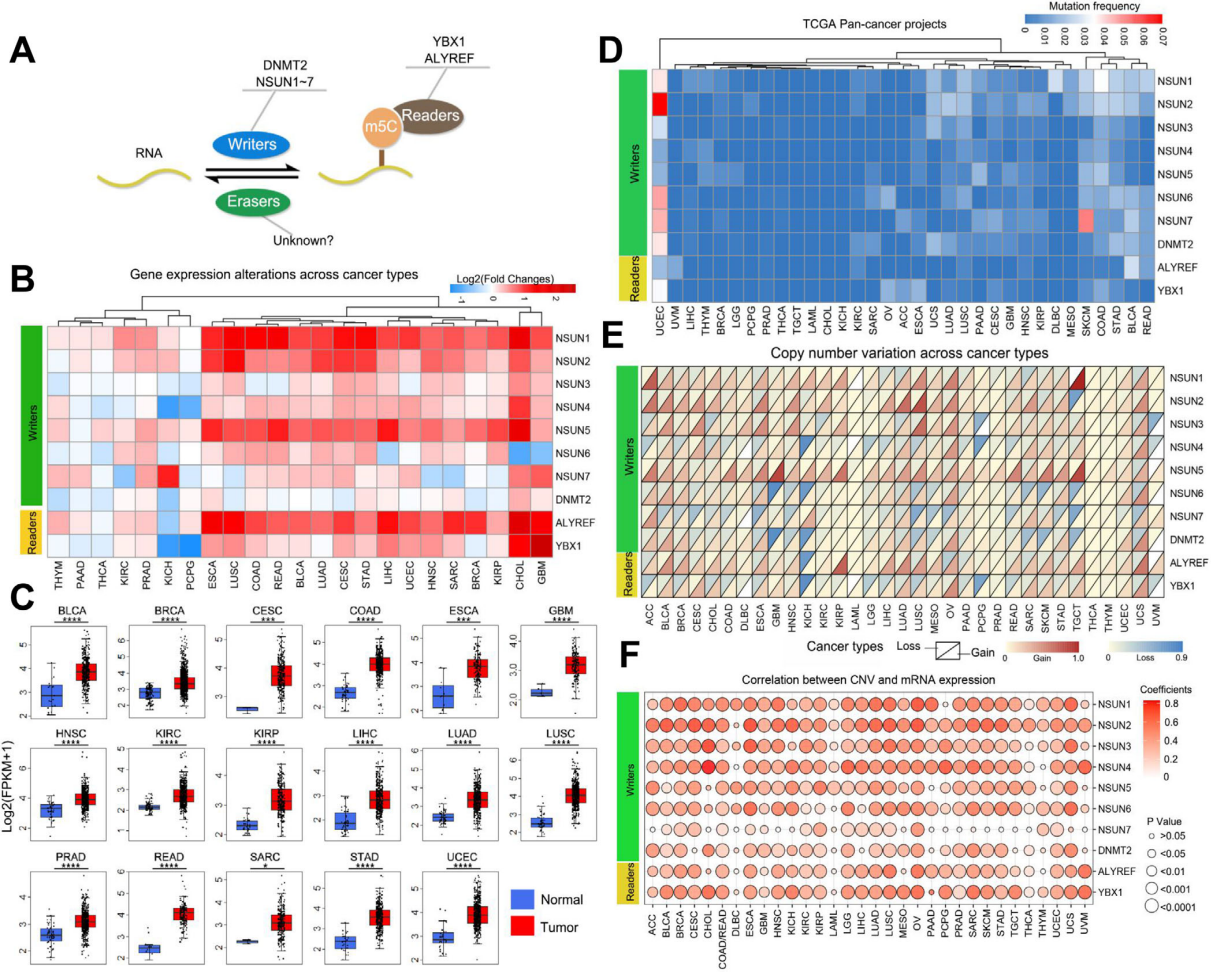
Genetic and expression alterations of m^5^C regulators in pan‐cancer. A, The writers, readers, and erasers diagram of m^5^C regulators. B, The gene expression alterations of m^5^C regulators across 23 cancer types selected from the TCGA database, fold changes are shown by a heat map, the upregulated genes are represented as red, and downregulated genes are represented as blue. C, The box diagrams showing NSUN1 expression across 17 cancer types from the TCGA database, and *t*‐test was used to calculate the significance level of differences by comparing tumor groups with normal groups. * *P*‐value < .05; **P‐value < .01; ***P‐value < .001; ****P‐value < .0001. D, The mutation frequency across 33 cancer types. The *x* axis indicates cancer types and *y* axis indicates m^5^C regulators. E, The CNV gain and loss frequency across 33 cancer types. The CNV gain frequency is colored by dark red; the CNV loss frequency is colored by midnight‐blue. The *x*‐axis indicates cancer types, and the *y*‐axis indicates m^5^C regulators. F, Correlation between CNV and mRNA expression across 33 cancer types from TCGA database. The point diagram is depicted to show the relationship, the size of a point is represented the *P*‐value, the correlation coefficient was colored by red; the greater the correlation, the deeper the red. The *x*‐axis indicates cancer types, and *y*‐axis indicates m^5^C regulators

We collected the data across 33 cancers from the TCGA database (Figure S1; Table S1) and selected 23 cancers with at least two normal controls to conduct differential expression analysis (Figure 1B). We found significant upregulation of four regulators (*NSUN1*, *NSUN2*, *NSUN5*, and *ALYREF*) in most cancers, whereas other regulators showed slight expression alterations. Meanwhile, unpaired *t*‐test analysis was performed to validate that these four regulators were significantly highly expressed in multiple cancers compared with normal controls from the TCGA database (Figure 1C; Figure S2A‐C). The GEO database with 17 tumor tissues demonstrates similar results (Figure S3A‐E). To explore the reasons leading to m^5^C regulator expression alterations, the mutation frequencies of regulators were first calculated and showed universally low in most TCGA cancers (Figure 1D; Table S2). The Cancer Cell Line Encyclopedia (CCLE) database containing 746 cell lines across 22 cancers represented similar results (Figure S4A). Meanwhile, we discovered that most writers showed higher mutation frequencies than readers (Figure S4B). Subsequently, we explored the copy number variation (CNV) of regulators across 33 TCGA cancers and found that CNV frequently occurred (Figure 1E; Table S3). The regulators (*NSUN1*, *NSUN2*, *NSUN5*, and *ALYREF*) with higher expression were accompanied by higher amplification frequencies. On the contrary, regulators (*NSUN7* and *DNMT2*) with lower expression appeared to have higher deletion frequencies. The results of the CCLE database showed similar CNV alterations (Figure S4C‐H; Table S4). Moreover, regulators (*NSUN1*, *NSUN2*, *NSUN3*, *NSUN5*, and *ALYREF*) with prevalent amplification frequencies represented a stronger correlation with their expression levels (Figure 1F and Figure S5; Tables S5 and S6), whereas *NSUN7* and *DNMT2*, with higher deletion frequencies, displayed a weaker association with their expression levels.

To further explore the molecular mechanisms of m^5^C regulators, we observed the correlation between regulators’ expression and 50‐hallmark‐related cancer pathway activities (Figure 2A; Table S7). We observed that *NSUN1*, *NSUN5*, *ALYREF*, and *YBX1* were positively correlated with MYC targets, DNA repair, G2M checkpoint, and E2F targets pathways. On the contrary, *NSUN1*, *NSUN5*, and *ALYREF* were negatively correlated with UV response, TGF‐β signaling, and protein secretion pathways. Furthermore, *NSUN3* and *YBX1* were positively correlated with more pathways; *NSUN6* and *NSUN7* showed negative correlations with more pathways (Figure 2B). To further analyze the cross‐talk of regulators, we analyzed the GSE133621 dataset, in which the gene expressions of wild‐type and *NSUN2*‐knockdown T24 cells were examined. We found that several regulators were downregulated in *NSUN2*‐knockdown T24 cells (Table S8). Meanwhile, pathways (TNF, JAK‐STAT, and cytokine signaling) and biological processes (cell death, apoptosis, and cytokine‐mediated signaling) were highly enriched in NSUN2‐knockdown T24 cohorts (Figure S6A‐D). Moreover, in pan‐cancer levels, several regulators (*NSUN1*, *NSUN2*, and *NSUN5*) showed co‐expression relationships, and their protein‐protein interaction (PPI) were prevalent, revealing that they might be affected by each other (Figure 2C and D; Table S9).

**FIGURE 2:**
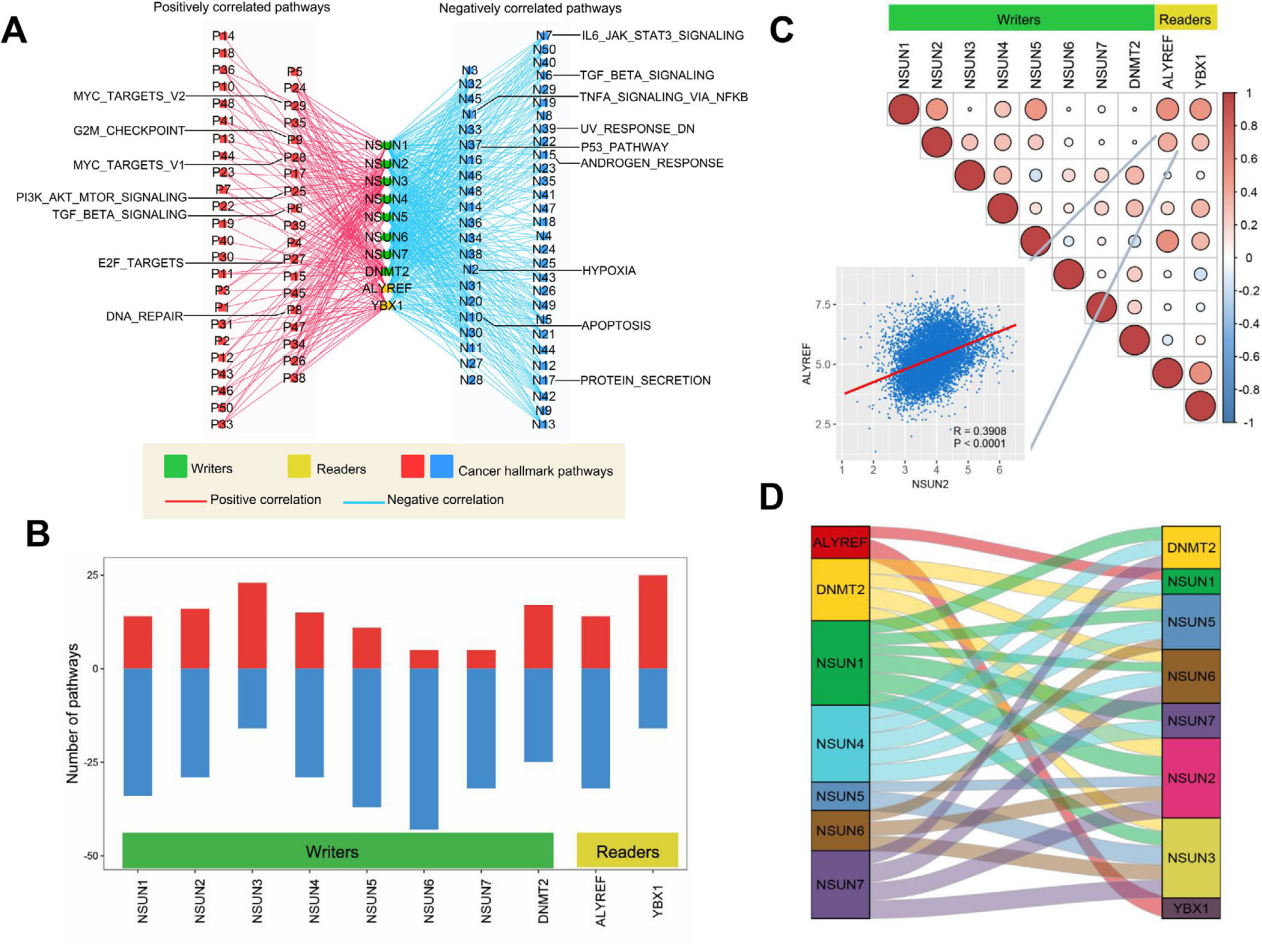
The correlation between cancer hallmark‐related pathways and m^5^C regulators. A, The network planning shows the positive or negative correlations between hallmark‐related cancer pathways and m^5^C regulators. Correlation with *P*‐value < .05 is selected. Positive correlations are shown by red; negative correlations are shown by blue; m^5^C writers are represented by green; m^5^C readers are represented by yellow. B, The bar diagrams show that the number of pathways is positively or negatively correlated with m^5^C regulators. The upper bar diagrams colored by red represented the number of positive correlations. The lower panel colored by blue represented the negative correlations. C, The correlation diagrams show the correlation among m^5^C regulators. The positive correlations are colored by red, and negative correlations are colored by blue. The size of the point represents the *P*‐value. D, The San‐key diagram shows the protein‐protein interaction among m^5^C regulators

We further excavated the regulators‐prognosis correlation utilizing the TCGA clinical data. We found that at least one regulator was correlated with the patients’ prognosis in 22 cancers (Figure 3A). Intriguingly, we observed that *NSUN1* represented high hazard ratios in many cancers, and patients with high *NSUN1* expression showed poorer survival probabilities across nine cancers (Figures S7 and S8). Other regulators, *NSUN2*, *NSUN5*, and *ALYREF*, also showed high hazard ratios in several cancers. Noticeably, *YBX1* had a slight expression alteration but still represented high hazard ratios in many cancers (Table S10). Moreover, more regulators were related to the prognosis of KIRC, LIHC, ACC, and LGG patients. In KIRC, some regulators (*NSUN1*, *NSUN2*, *NSUN5*, *NSUN6*, *ALYREF*, and *YBX1*) showed risky functions, and others showed protective functions (Figure S9A). However, almost all regulators acted as an oncogene in LIHC, LGG, and ACC (Figure S9B and C). For these four cancers, we further conducted unsupervised clustering and stratified them into two subtypes separately (Figure 3B; Figure S10A‐C). The survival rates of the two subtypes in different cancers were dramatically different, demonstrating consensus clustering of m5C regulators could be a suitable prognosis‐stratification method (Figure 3C; Figure S11A‐C). To further understand the clinical relevance of m^5^C regulators, we established a PPI network between regulators and 123 clinical‐related cancer genes.^5^ We found that several regulators interacted with different clinical‐related genes. YBX1 and DNMT2, and NSUN1 and NSUN2, interacted with the same genes, MYC and NPM1. The two readers also interacted with the same gene, AKT1 (Figure S12; Table S11).

**FIGURE 3:**
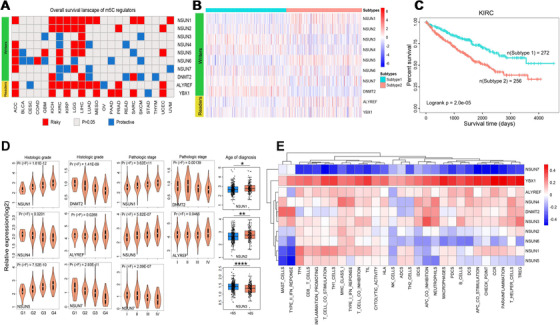
The relationships between m^5^C regulators and clinics. A, The correlation between m^5^C regulators and overall survival across 22 cancer types with at least one regulator related to prognosis. Red represents the higher expressions of m^5^C regulators that are significantly correlated with poorer survival; blue represents the higher expressions of m^5^C regulators that are significantly related to better survival. *P*‐value > .05 is colored by gray. B, The heat map shows the subgroups identified via a global expression pattern of m^5^C regulators in KIRC. C, Kaplan‐Meier survival curves of patients grouped by the global expression pattern of m^5^C regulators in KIRC. The log‐rank test *P*‐value is shown. D, The violin diagrams and box plots showing the correlation between m^5^C regulators and histologic grade, pathologic stage, and the patients' age. One‐way analysis of variance (ANOVA) was used to compare the differences of three and more groups. *P*‐value < .05 is regarded as significant. The *x*‐axis indicates clinical information, and the *y*‐axis indicates the RNA expression of m^5^C regulators. E, The correlation between the activity scores of immune‐related gene signatures and the expression of m^5^C regulators. The positive correlation is colored by red; the negative correlation is colored by blue. The greater the correlation, the deeper the color. The *x*‐axis indicates cancer types, and the *y*‐axis indicates m^5^C regulators

Because nine regulators were associated with the prognosis of KIRC patients, we finally focused on KIRC. We compared the activity of Gene Oncology (GO) and Kyoto Encyclopedia of Genes Genomes (KEGG) pathways based on two KIRC subtypes (Table S12, Figure S13A‐D). We discovered that cell component dysregulations mainly occurred in multiple enzyme complexes and some membrane sites. The disorders of molecular functions were some changes in enzyme activities and molecular bindings. The alterations of biological processes principally contained membrane transports and tRNA‐5‐leader removal. The alterations of KEGG were mostly correlated with multiple cancer pathways. Furthermore, regulators showed different expressions in various grades, stages, and age groups (Figure 3D). Intriguingly, *NSUN7* expression showed a universal negative correlation with 29 immune‐related gene sets, while *YBX1* represented an opposite result (Figure 3E; Table S13). Some higher expressions of risky regulators (*NSUN1*, *ALYREF*, and *YBX1*) were accompanied by increased immune infiltration levels. Conversely, the protective regulator *NSUN7* showed lower expression in increased immune infiltration circumstances (Figure S14). Finally, we discovered different immune‐related gene signatures that showed high or low risks to the KIRC patients’ prognosis (Table S14). Meanwhile, combining the KIRC immune‐related gene signatures and subtypes was a feasibility method to predict KIRC patients’ prognosis (Figure S15).

In summary, our study highlights that the roles of m^5^C regulators in pan‐cancer and provides a foundation for the development of therapeutic strategies based on RNA methylation. We expect these organized information and hypotheses will be mined and validated by other researchers over time.

## CONFLICT OF INTEREST

The authors declare there is no conflict of interest.

## CONSENT FOR PUBLICATION

All the authors consent for publication.

## AUTHOR CONTRIBUTIONS

Zhihong Zhang and E. Du conceived of the project. E. Du and Jingxian Li designed and performed the research with contributions from Zhihong Zhang, Shuai Li, Fei Sheng, and Jianqiang Zhu. Jingxian Li and Fei Sheng provided constructive feedback and constructed the web‐based resource. Jingxian Li and Shuai Li analyzed the data. Zhihong Zhang and E. Du supervised research and provided critical advice on the study. Jingxian Li and E. Du wrote the manuscript, with input from other co‐authors.

## Supporting information

Supporting Information

Supporting Information

## Data Availability

All publicly available data referenced herein can be retrieved from TCGA (https://portal.gdc.cancer.gov/), GEO (https://www.ncbi.nlm.nih.gov/gds/), UCSC Xena (https://xena.ucsc.edu/), CCLE (https://portals.broadinstitute.org/ccle), STRING (https://string-db.org/).
