# Supplementary material for: A pan‐cancer analysis reveals genetic alterations, molecular mechanisms, and clinical relevance of m5C regulators
Source: Clin Transl Med. 2020 Sep 15;10(5):e180. doi: 10.1002/ctm2.180 (PMC7507430; doi:10.1002/ctm2.180)
Supplement: Supplementary file 2 — Supporting Information [file CTM2-10-e180-s002.pdf]

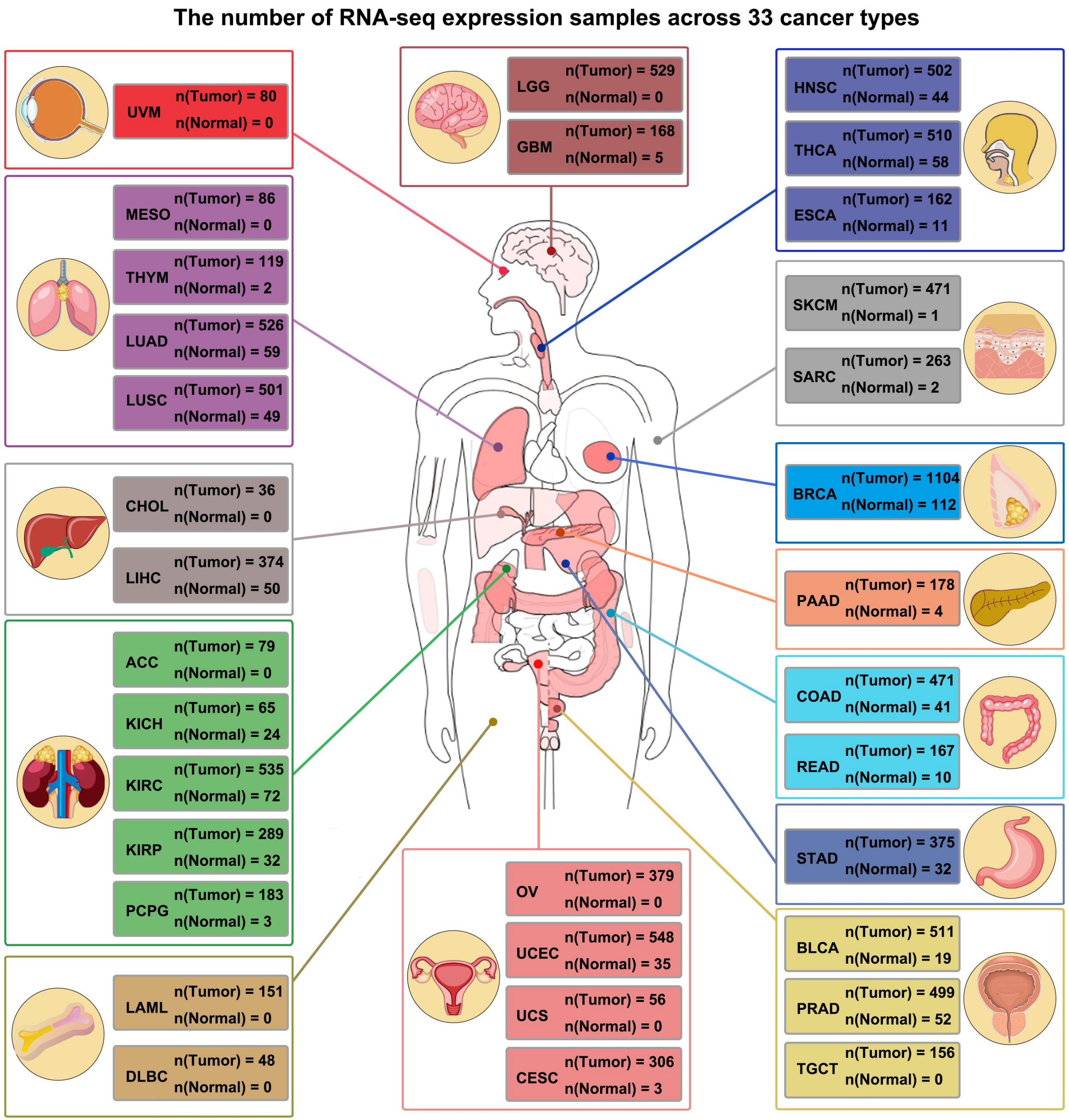

Supplementary Fig. 1. The distributions and samples' number of 33 cancer types from TCGA database in human body.

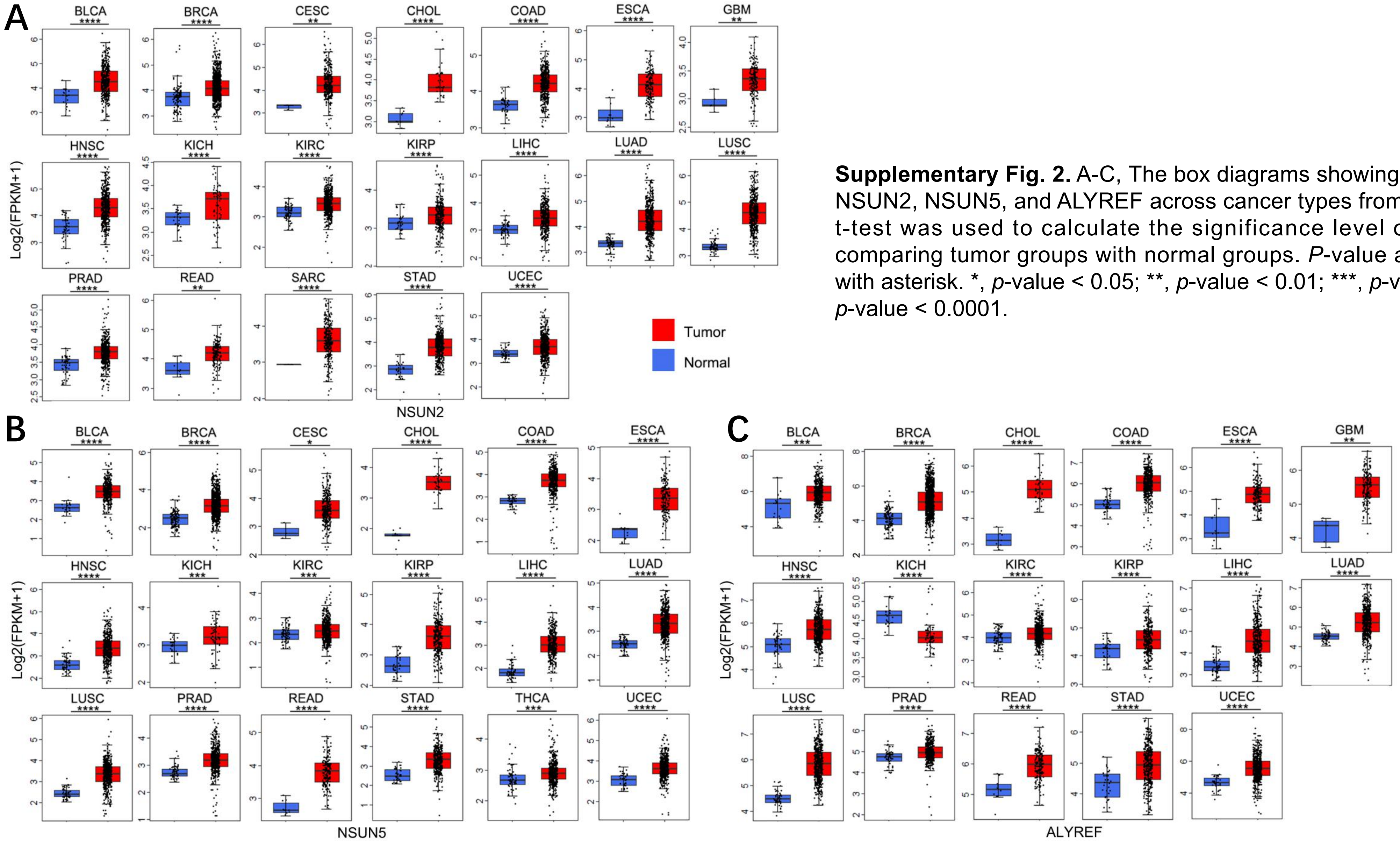

Supplementary Fig. 2. A-C, The box diagrams showing the expression of NSUN2, NSUN5, and ALYREF across cancer types from TCGA database, t-test was used to calculate the significance level of differences by comparing tumor groups with normal groups. *P*-value are also identified with asterisk. *, *p*-value < 0.05; **, *p*-value < 0.01; ***, *p*-value < 0.001; ****, *p*-value < 0.0001.

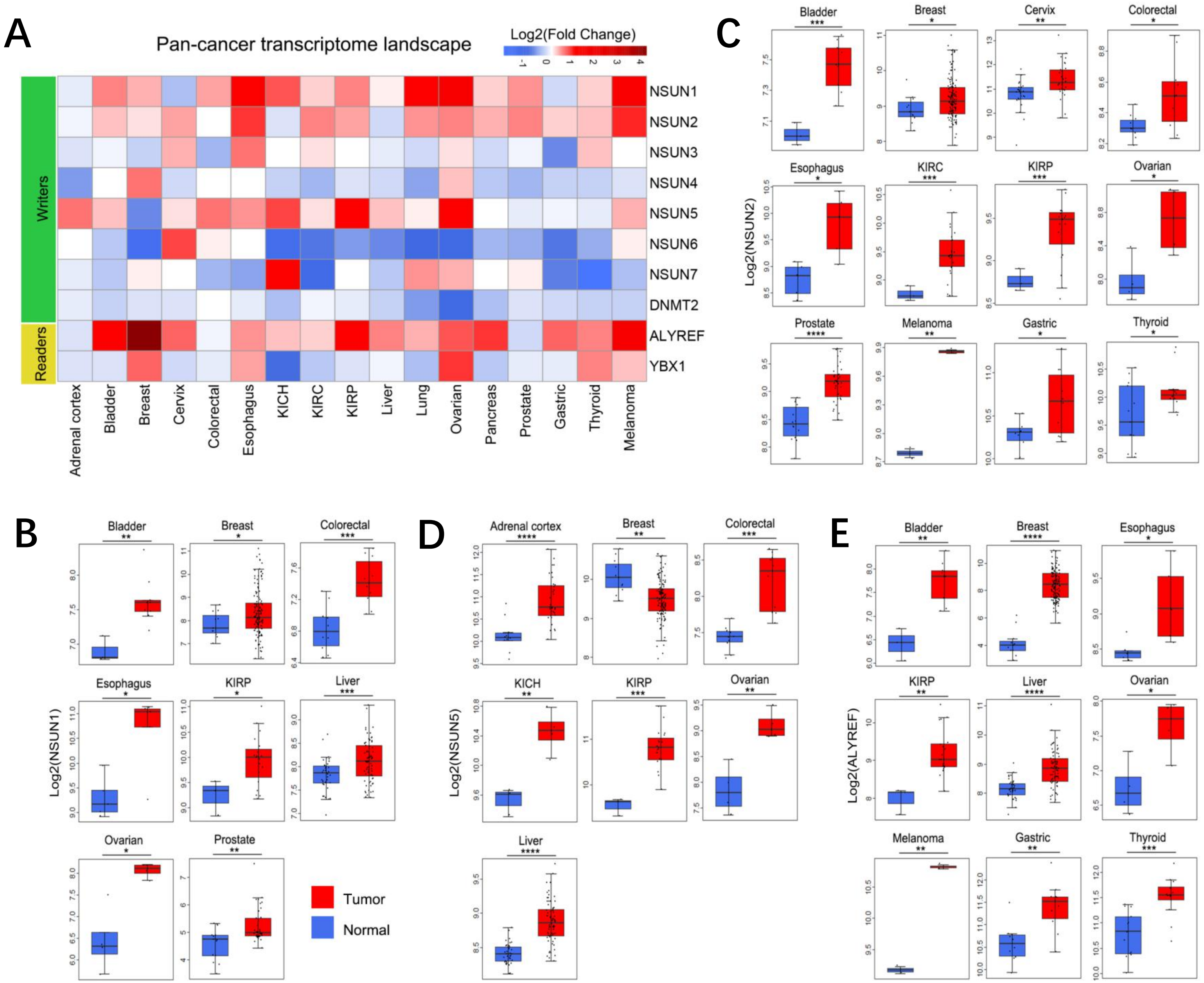

Supplementary Fig. 3. Expression alterations of m5C regulators across cancer types. A, The expression alterations of m5C regulators across 17 cancers from the GEO database. The heatmap was depicted to shown the Log2(Fold Change). The upregulated genes were colored by red, the downregulated genes were colored by blue. B, The boxplot showed the expression of NSUN1 across 8 cancer types, t-test was used to calculate the significance level by comparing tumor groups with normal groups. *P*-value are also identified with asterisk. *, *p*-value < 0.05; **, *p*-value < 0.01; ***, *p*-value < 0.001; ****, *p*-value < 0.0001.

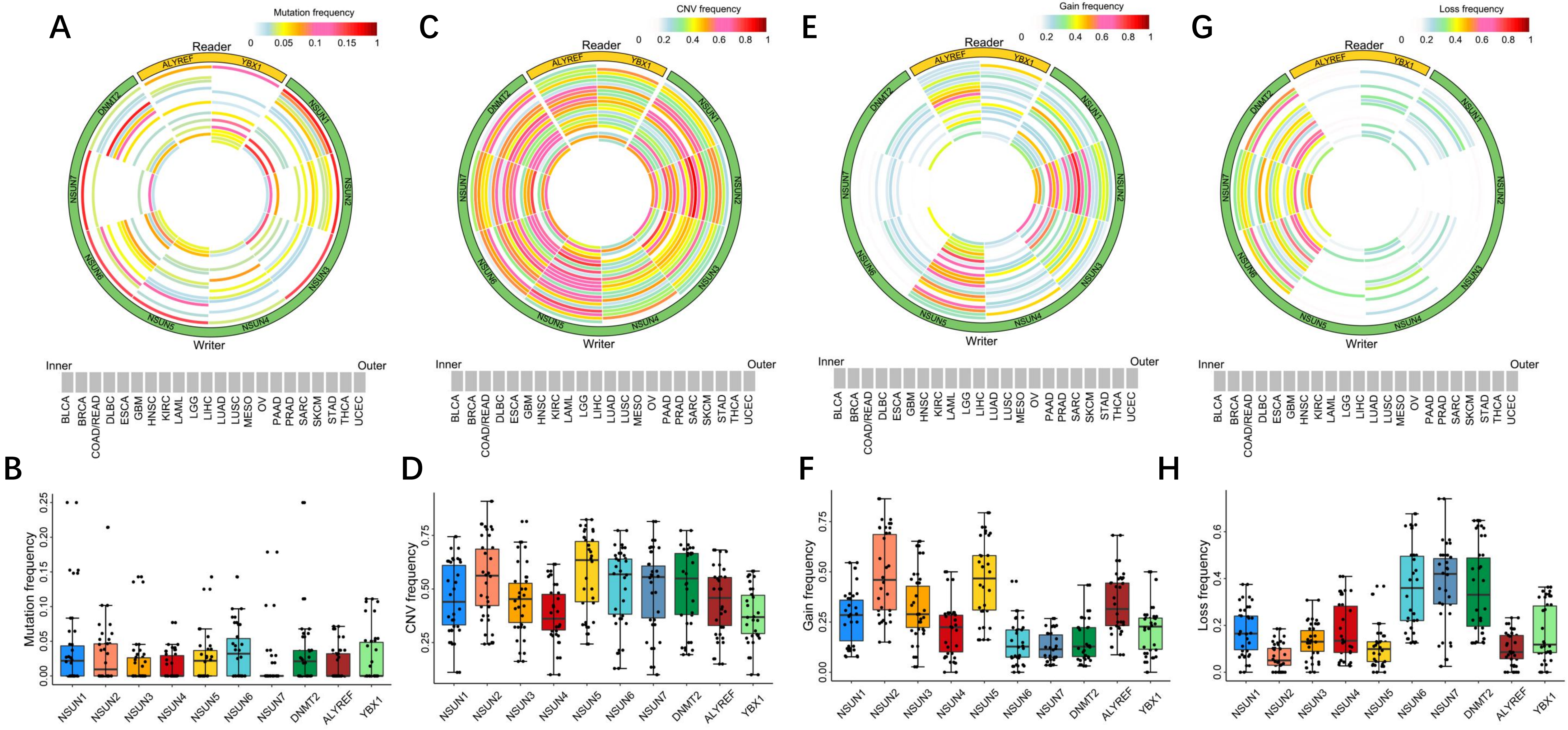

Supplementary Fig. 4. The frequencies of mutation, CNV, amplification and deletion across cancer types. A, C, E, G, The circos plots were portrayed to represent mutation, CNV, amplification and deletion frequencies across 22 cancer types dowloaded from CCLE database. B, D, F, H, The boxplot were depicted to show the mutation, CNV, Gain and Loss frequency across 22 cancer types in defferent m5C regulators.

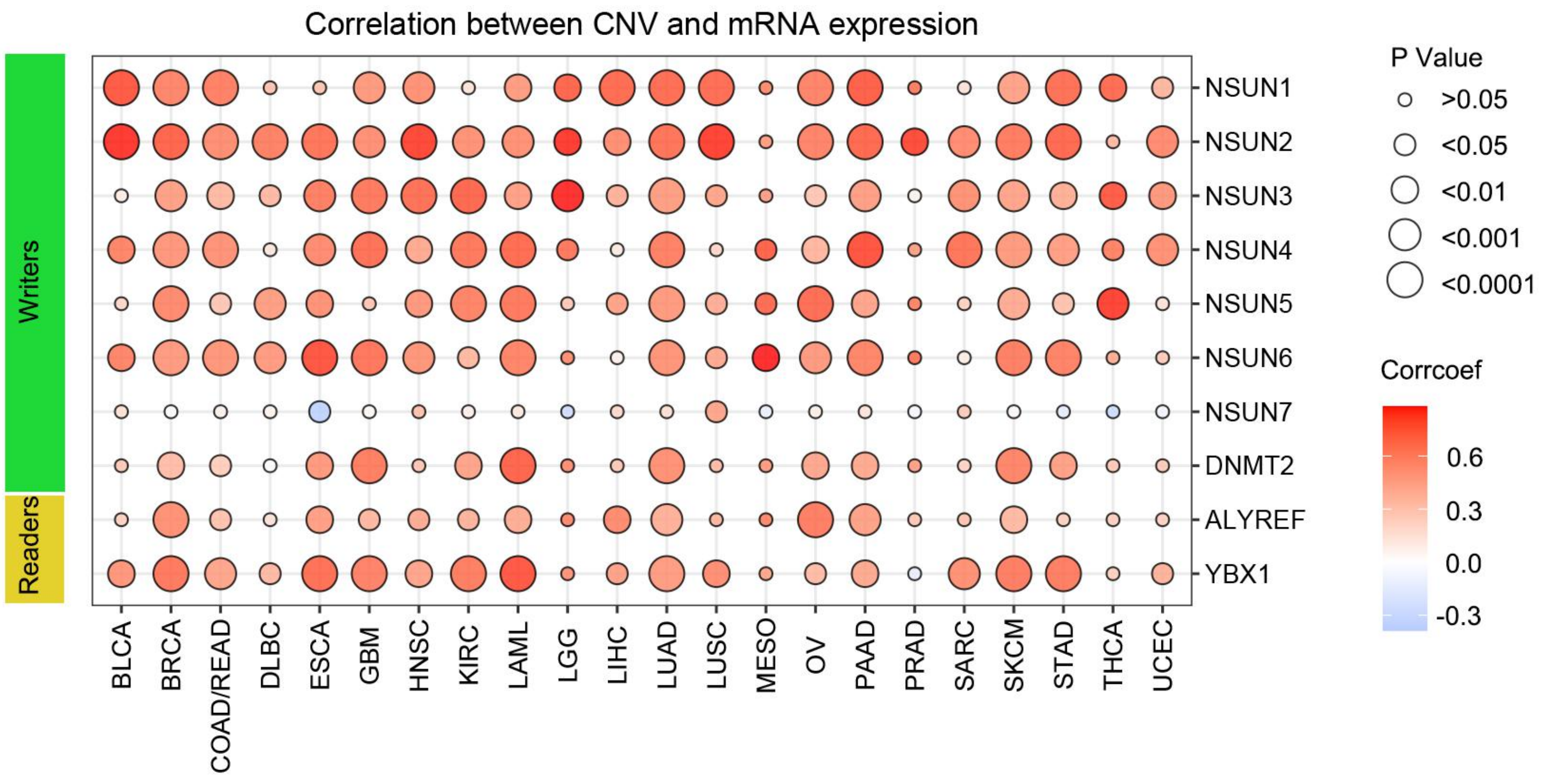

Supplementary Fig. 5. Correlation between CNV and expression of m5C regulators in CCLE database. The pearson correlation coefficients between CNV and mRNA expression were visualized by correlogram. The size of point represented different p-value, positive correlations were colored by red, and negative correlations were colored by blue.

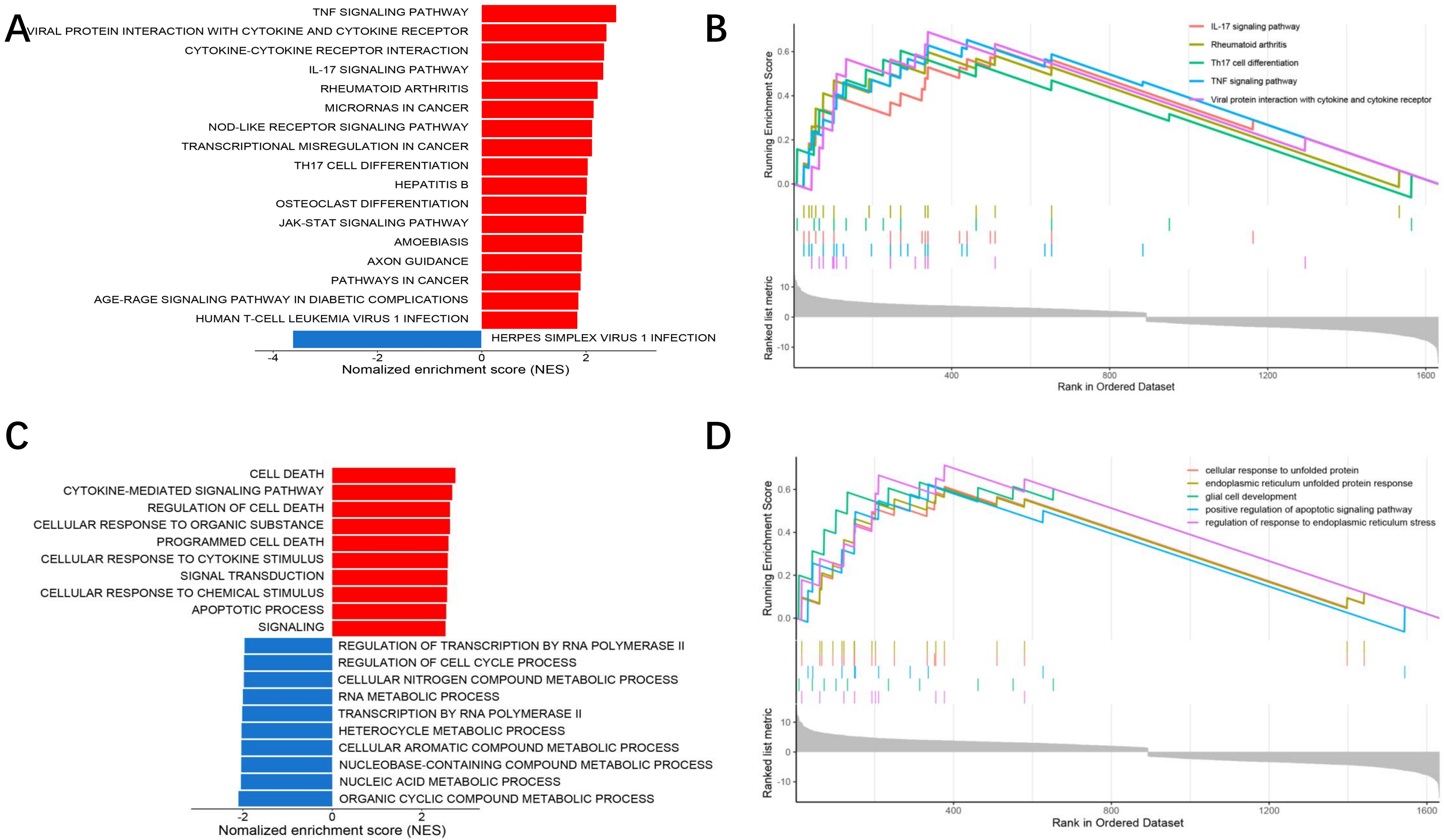

Supplementary Fig. 6. GSEA-enrichment analyses of pathways and biological processes. A, Distribution of normalized enrichment scores for pathways. B, GSEA-enrichment plot of top 5 pathways. C, Distribution of normalized enrichment scores for biological processes. D, GSEA-enrichment plot of top 5 biological processes.

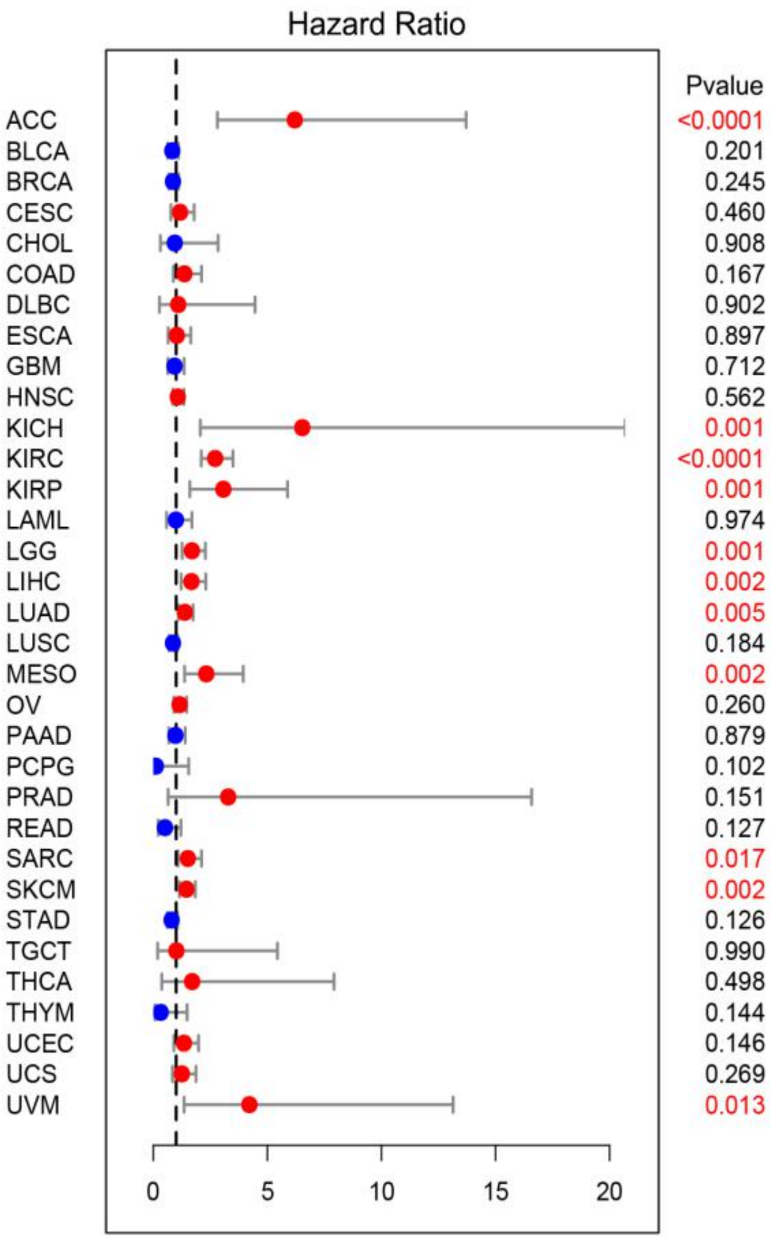

Supplementary Fig. 7. The distribution of NSUN1 hazard ratios across 33 cancer types. The red point indicates the high hazard ratio, and the blue point indicates the low hazard ratio. *P*-value < 0.05 is regarded as significant and identified by the red font.

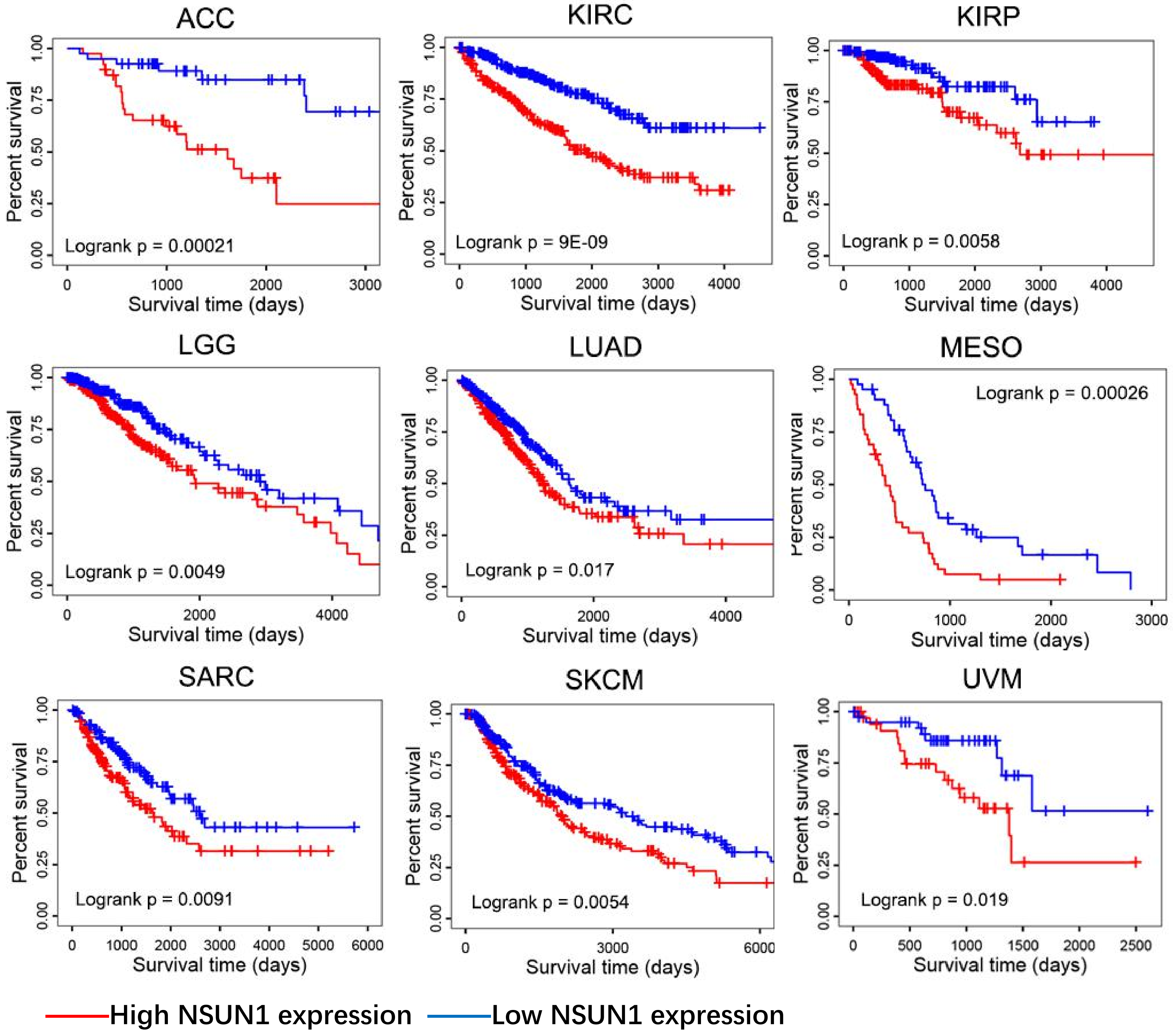

Supplementary Fig. 8. Kaplan-Meier survival curves of patients grouped by the expression of NSUN1 in individual cancer. The *p*-values calculated by log-rank test are shown.

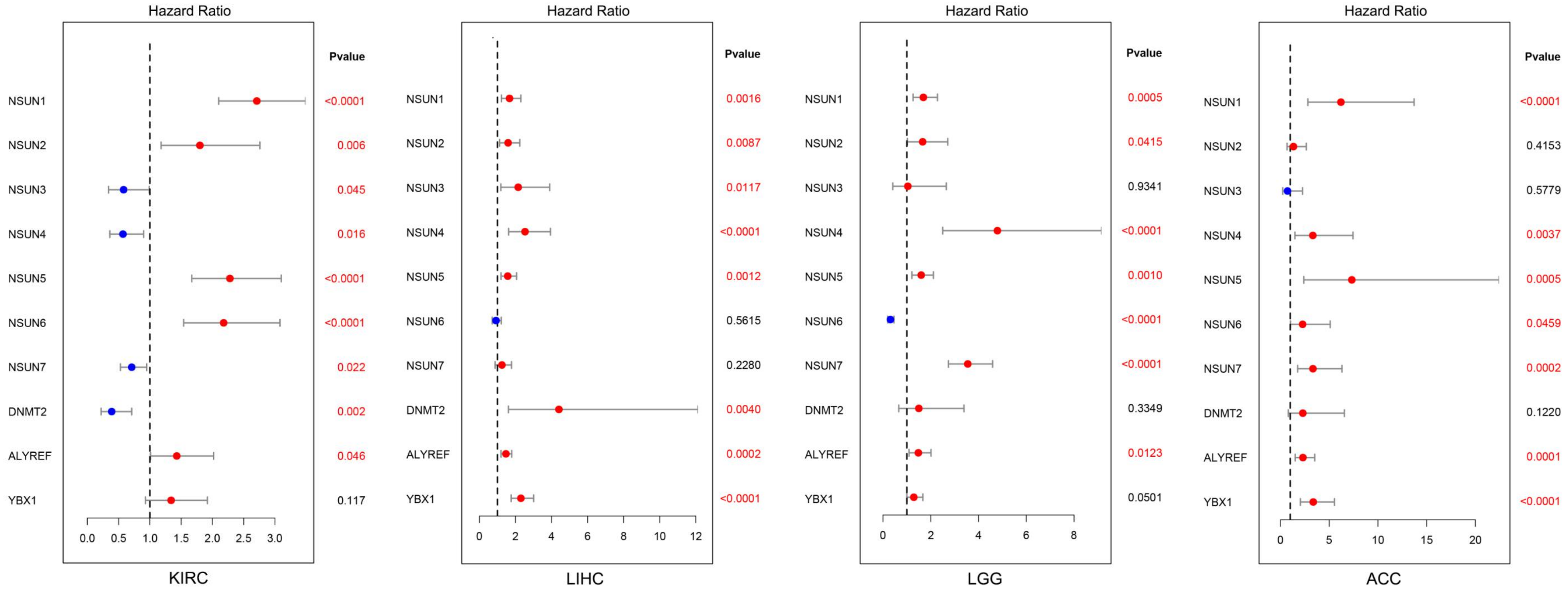

Supplementary Fig. 9. The distributions of individual m5C regulators' hazard ratios. A-D, The hazard ratios (HR) of all m5C regulators in KIRC, LIHC, LGG and ACC. The high hazard ratio (HR >1) were colored by red, the low hazard ratio (HR <1) were colored by blue.

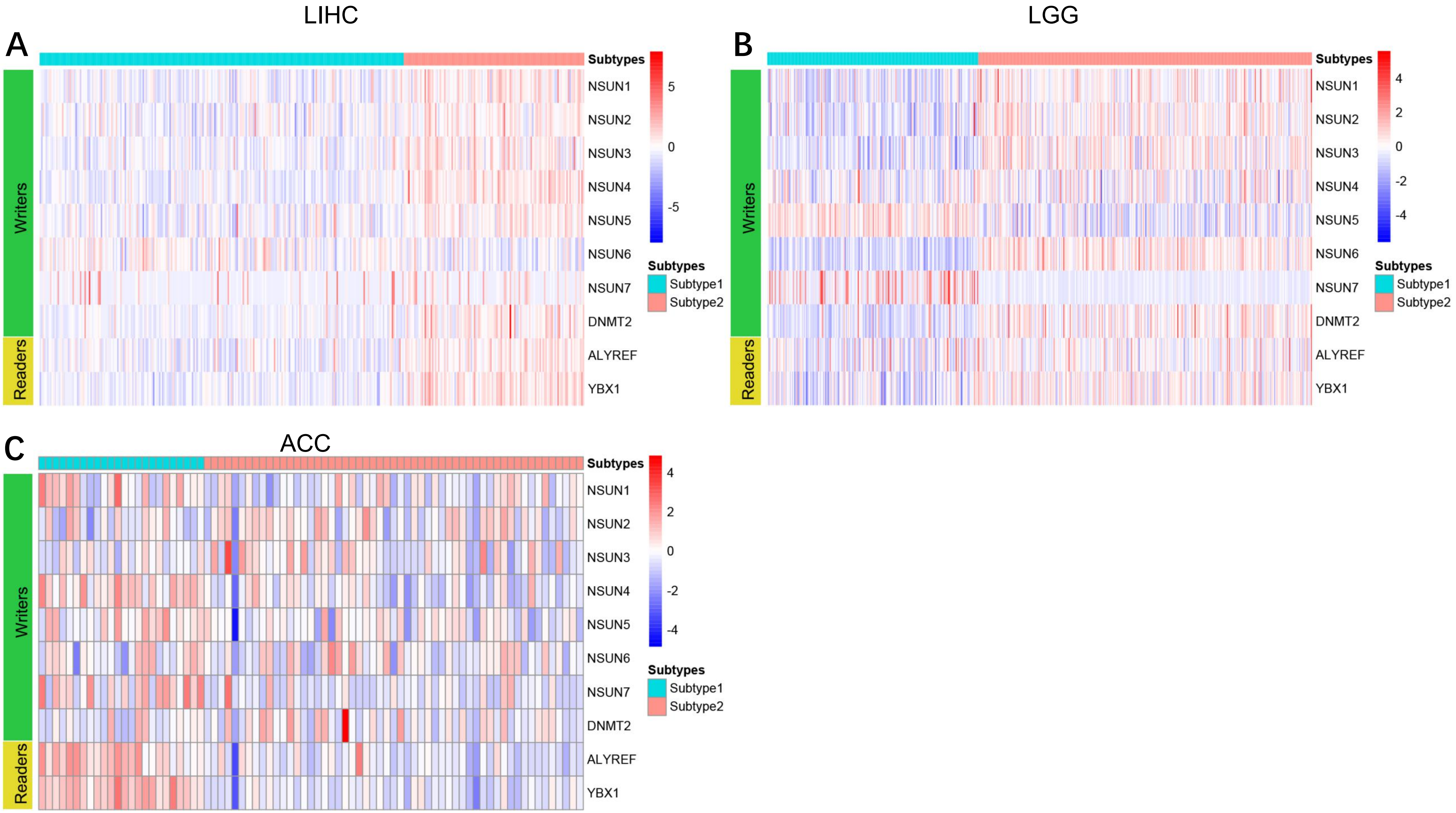

Supplementary Fig. 10. Heatmaps depicted by subgroups. A-C, The subgroups identified via global expression pattern of m5C regulators in LIHC, LGG and ACC. Euclidean distances were calculated and ward. D2 method were used to perform hierarchical clustering.

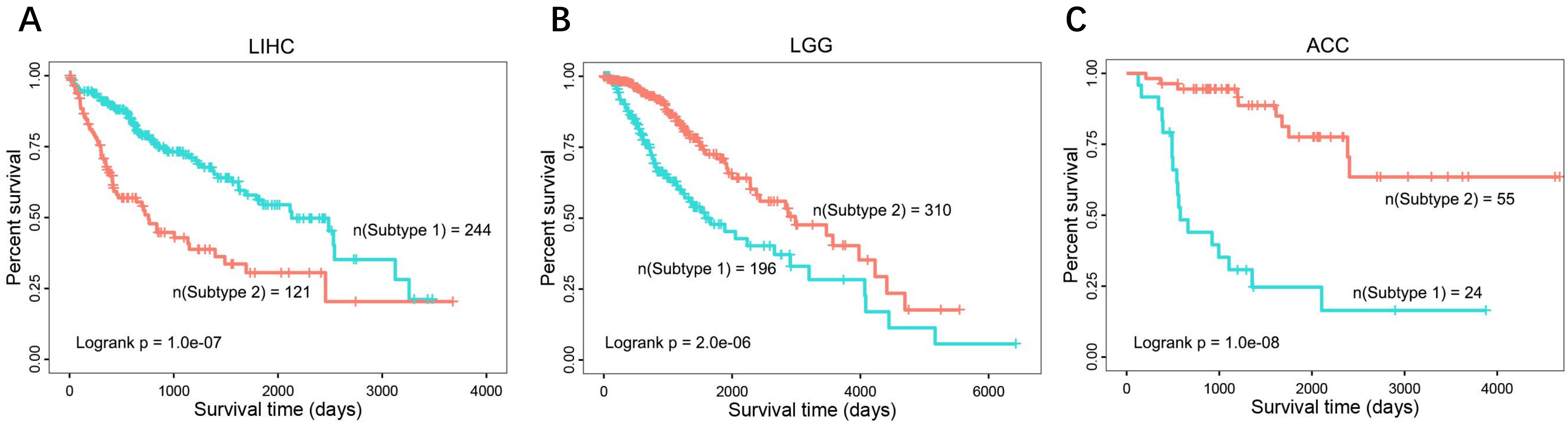

Supplementary Fig.11. Survival curves based on subgroups across cancer types. A-C, Kaplan-Meier survival curves of patients grouped by the global expression pattern of m5C regulators in LIHC, LGG and ACC. The *p*-values calculated by log-rank test are shown.

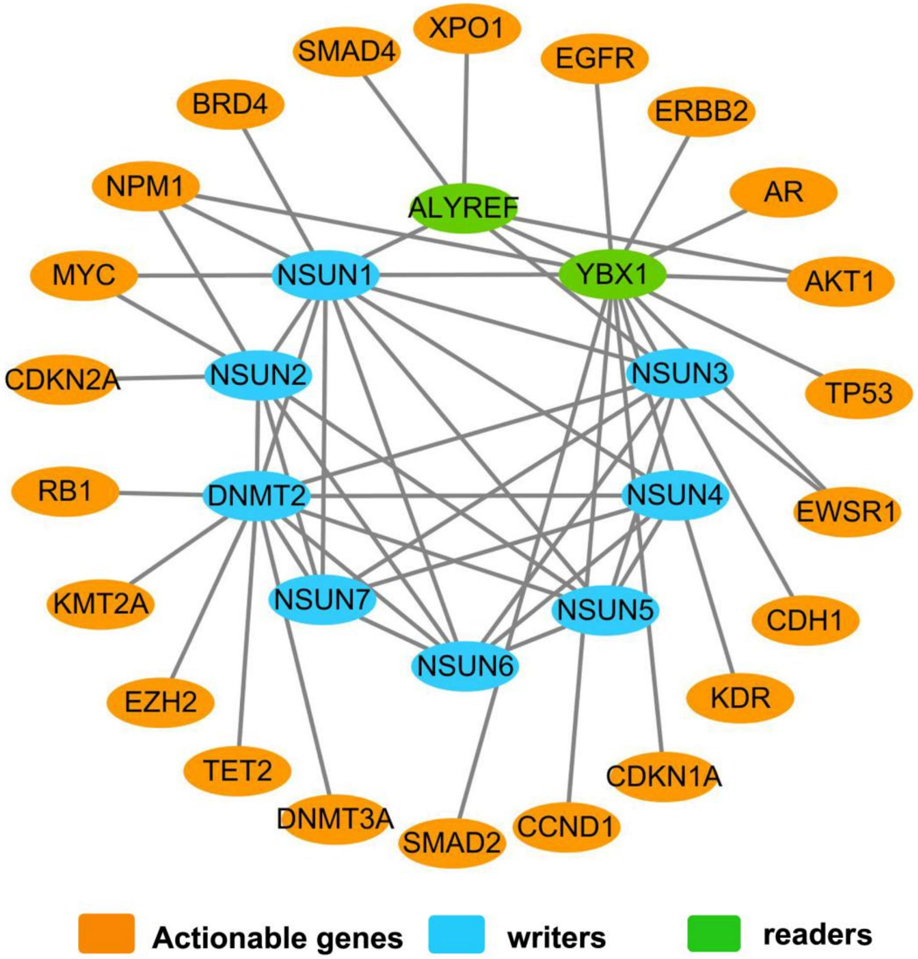

Supplement Fig. 12. The protein-protein interaction network between m5C regulators and clinically relevant cancer genes, obtained from STRING database and visualized by cytoscape software. Clinical cancer genes are colored by yellow, writers of m^5C are colored by blue, and readers of m5C are colored by green.

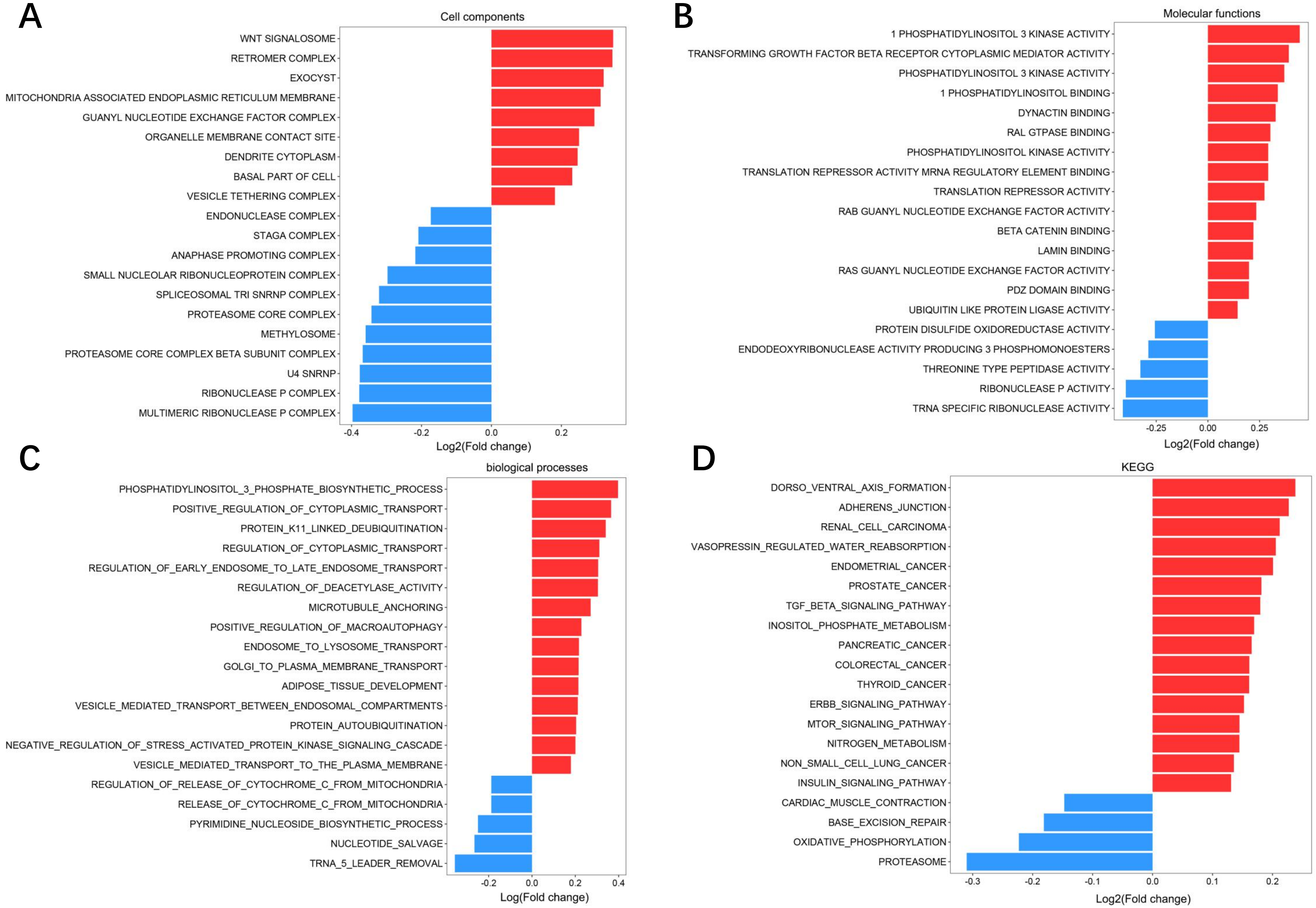

Supplement Fig. 13. Changes of Gene Ontology and KEGG. A-D. The barplots showed top 20 activity alteration of cell components, molecular functions, biological processes and KEGG pathways via comparing subtype2 with subtype1. The upregulation of activity were colored by red, and the downregulation of activity were colored by blue.

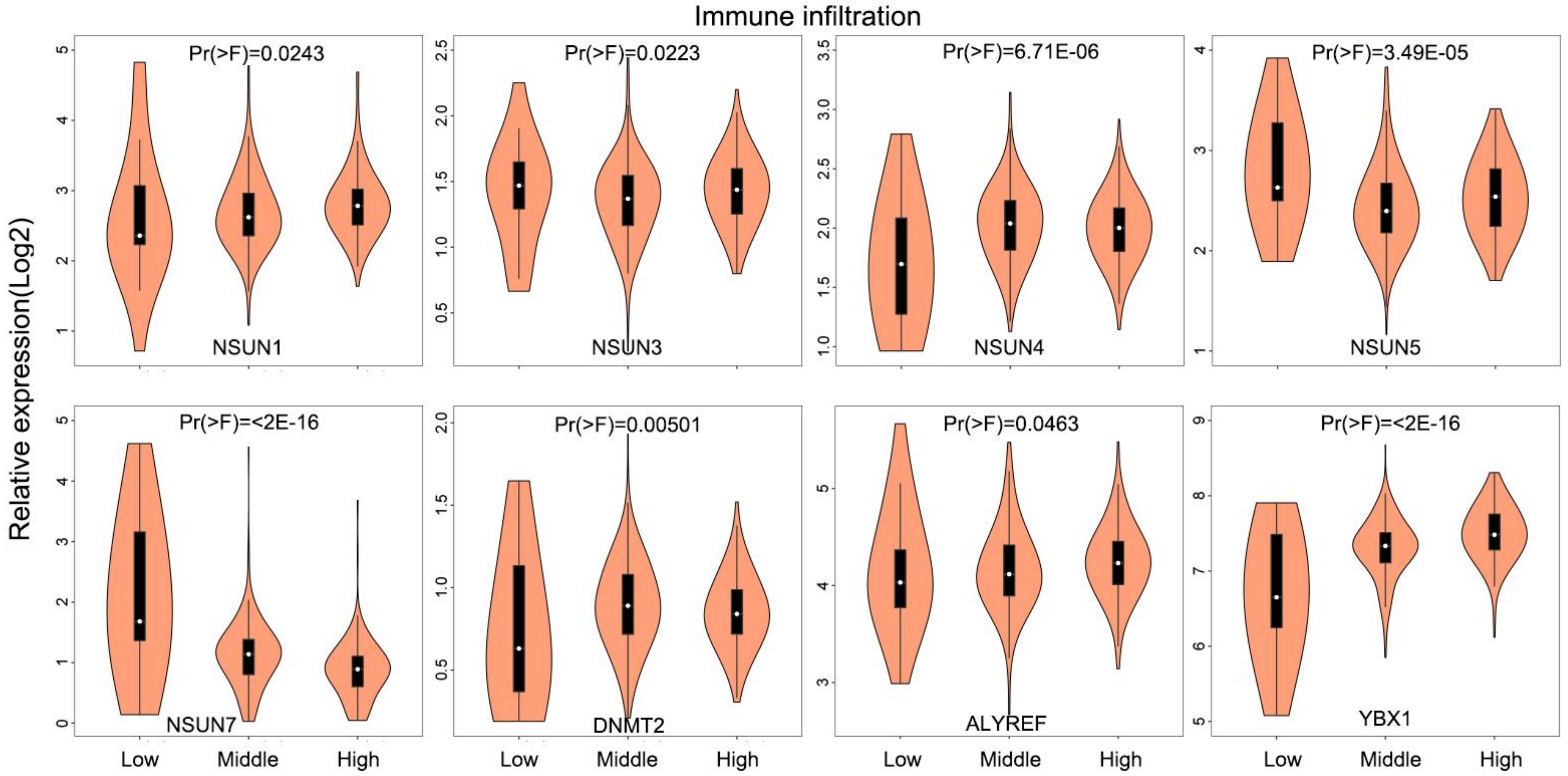

Supplement Fig. 14. The violin diagrams showing correlations between m5C regulators and the degrees of immune infiltration identified by activity scores of immune cells and immune response.

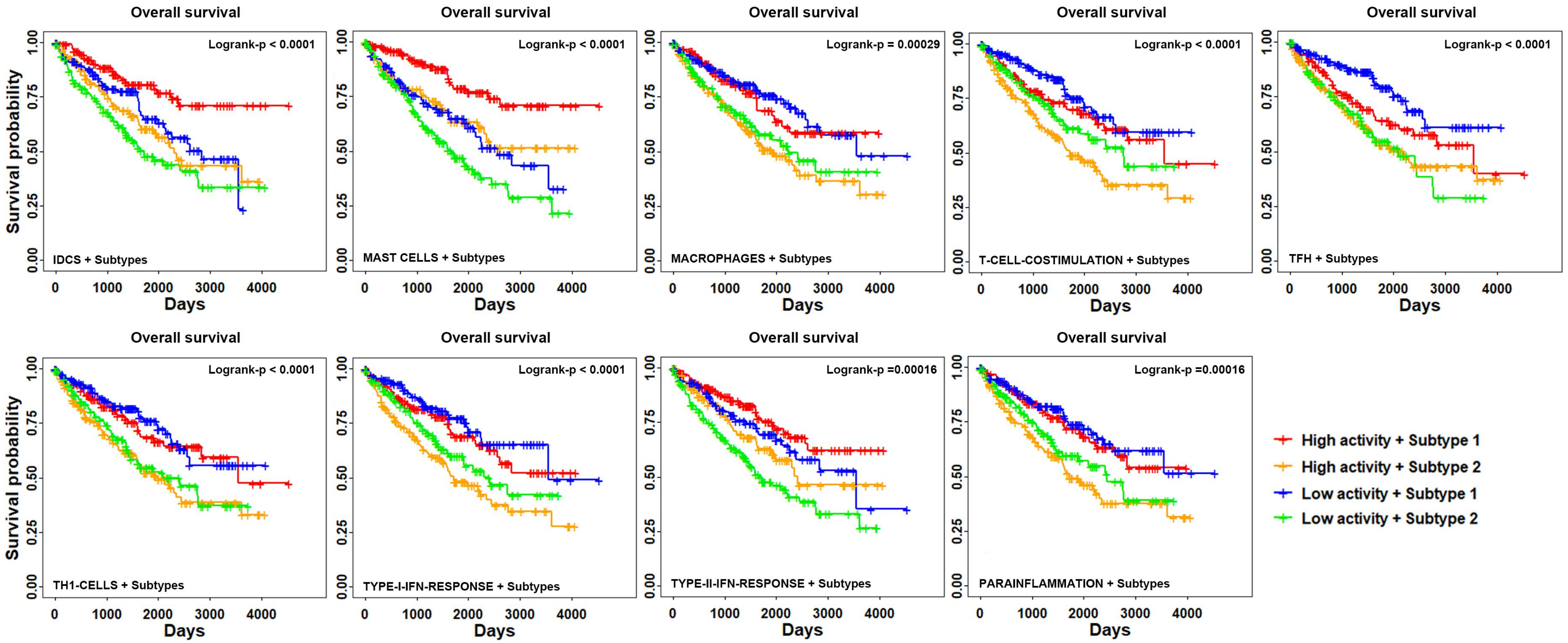

Supplementary Fig. 15. Survival curves analyzed by activities of immune cells or immune responses bind to subtypes based on expression of m⁵C regulators in KIRC. High activity binds subtype1 is colored by red, high activity binds subtype2 is colored by orange, low activity binds subtype1 is colored by blue, low activity binds subtype2 is colored by green. The *p*-values calculated by log-rank test are shown.
